# Clinical complete response and predictive factors in HER2-positive early breast cancer treated with neoadjuvant chemotherapy aimed at omission of surgery: an exploratory analysis of the JCOG1806 trial

**DOI:** 10.1007/s10147-026-02967-7

**Published:** 2026-01-22

**Authors:** Hideo Shigematsu, Tomomi Fujisawa, Fumikata Hara, Hiroji Iwata, Toshiyuki Ishiba, Yukinori Ozaki, Takehiko Sakai, Yasuaki Sagara, Akihiko Shimomura, Kazuki Sudo, Kaori Terata, Yoichi Naito, Kazuki Nozawa, Keita Sasaki, Noriko Mitome, Ryo Sadachi, Taro Shibata, Tadahiko Shien

**Affiliations:** 1 Department of Surgical Oncology, Research Institute for Radiation Biology and Medicine, Hiroshima University Hospital, 1-2-3-Kasumi, Minami-Ku, Hiroshima, 734-8551 Japan; 2 Gunma Prefectural Cancer Center, Gunma, Japan; 3 Aichi Cancer Center, Aichi, Japan; 4 Graduate School of Medical Sciences, Nagoya City University, Nagoya, Japan; 5 Institute of Science Tokyo Hospital, Tokyo, Japan; 6 Cancer Institute Hospital of the Japanese Foundation for Cancer Research, Tokyo, Japan; 7 Hakuaikai Sagara Hospital, Kagoshima, Japan; 8 National Center for Global Health and Medicine, Tokyo, Japan; 9 National Cancer Center Hospital, Tokyo, Japan; 10 Akita University Hospital, Akita, Japan; 11 National Cancer Center Hospital East, Kashiwa, Chiba Japan; 12 Japan Clinical Oncology Group Data Center/Operations Office, National Cancer Center Hospital, Tokyo, Japan; 13 Okayama University Hospital, Okayama, Japan

**Keywords:** Breast cancer, Primary systemic therapy, HER2, cCR, Omission of surgery

## Abstract

**Purpose:**

The JCOG1806 trial (jRCTs031190129) is underway to evaluate the omission of surgery in patients with human epidermal growth factor receptor (HER2)-positive early breast cancer who have a clinical complete response (cCR) after primary systemic therapy (PST). We aimed to assess the cCR rate in this trial and identify predictive factors.

**Methods:**

HER2-positivity was defined as an immunohistochemistry (IHC) score of 3 + or in situ hybridization-positivity. A cCR was defined as the absence of detectable lesions upon palpation, contrast-enhanced magnetic resonance imaging, and ultrasonography; biopsy-based confirmation was optional in hormone receptor (HR)-negative cases and mandatory in HR-positive cases. Multivariate logistic regression analyses were used to identify predictors of a cCR.

**Results:**

The cCR rate was 57.6% (196/340 patients; 95% confidence interval [CI]: 52.2–63.0%). Strongly estrogen-receptor (ER)-positive tumors (≥ 10%) were significantly less likely to have a cCR than ER-negative tumors (odds ratio [OR], 0.41; 95% CI: 0.20–0.81). IHC 3 + tumors had higher cCR rates than IHC 1 + or 2 + tumors (OR, 2.19; 95% CI: 1.01–4.74). Compared with histological grade I tumors, cCR odds were higher in grade II (OR: 2.92; 95% CI: 1.07–7.93) and III (OR: 4.90; 95% CI: 1.76–13.7) tumors. Among patients without a cCR patients undergoing surgery, 22.2% were diagnosed with ypT0 tumors upon analysis of surgical specimens.

**Conclusion:**

ER-negativity, an IHC score of 3 + , and a higher histological grade were independent predictors of a cCR. Identifying these features may improve the feasibility and safety of surgery omission for patients with HER2-positive early breast cancer.

**Supplementary Information:**

The online version contains supplementary material available at 10.1007/s10147-026-02967-7.

## Introduction

Primary systemic therapy (PST) is the standard treatment for human epidermal growth factor receptor 2 (HER2)-positive breast cancer, supported by high pathological complete response (pCR) rates and the ability to guide adjuvant therapy based on residual disease [1–4]. Currently, the combination of trastuzumab and pertuzumab with chemotherapy is the standard PST regimen, yielding pCR rate of 40–70% [5–7].

In this context, the possibility of omitting surgery in patients who have an excellent response to PST has gained increasing attention [8–10]. Kuerer et al. demonstrated via a phase II trial that omission of surgery may be feasible in patients with HER2-positive or triple-negative breast cancer who have a pCR upon PST, as confirmed via image-guided, vacuum-assisted biopsy, yielding favorable local control and survival outcomes [11, 12]. The OPTIMIST trial, a non-inferiority study, is being conducted to further evaluate this strategy in patients with HER2-positive, triple-negative, or estrogen receptor (ER)-low-positive tumors by using stringent magnetic resonance imaging (MRI) and biopsy-based eligibility criteria [13]. These studies suggest that omission of surgery may be a viable treatment option for selected patients.

However, as eligibility was assessed only after PST in those two studies, the proportion and characteristics of patients who may qualify before treatment remain unclear. Although clinical complete response (cCR) rates were reported in previous studies based on the response evaluation criteria in solid tumors, those criteria differ from those used to define eligibility for surgery omission, limiting their relevance in this context [14–16]. Thus, the appropriateness of extrapolating those findings to the feasibility of surgery omission is unclear.

To address these clinical questions, we conducted an exploratory analysis of the JCOG1806 trial, a single-arm, confirmatory phase III study of the feasibility of surgery omission in patients with cT1–2N0M0, HER2-positive early breast cancer [10]. Eligible patients received PST, and surgery was not performed in those with a cCR. Unlike other studies, patients were enrolled before treatment initiation in the JCOG1806 trial, allowing for a comprehensive analysis of the proportion and characteristics of patients suited for surgery omission. In this exploratory analysis, we focused on the cCR rate—a prerequisite for consideration of surgery omission—and investigated clinicopathological factors that may be predictive of a cCR in patients with HER2-positive early breast cancer.

## Materials and methods

### Study design

**Fig. 1:**
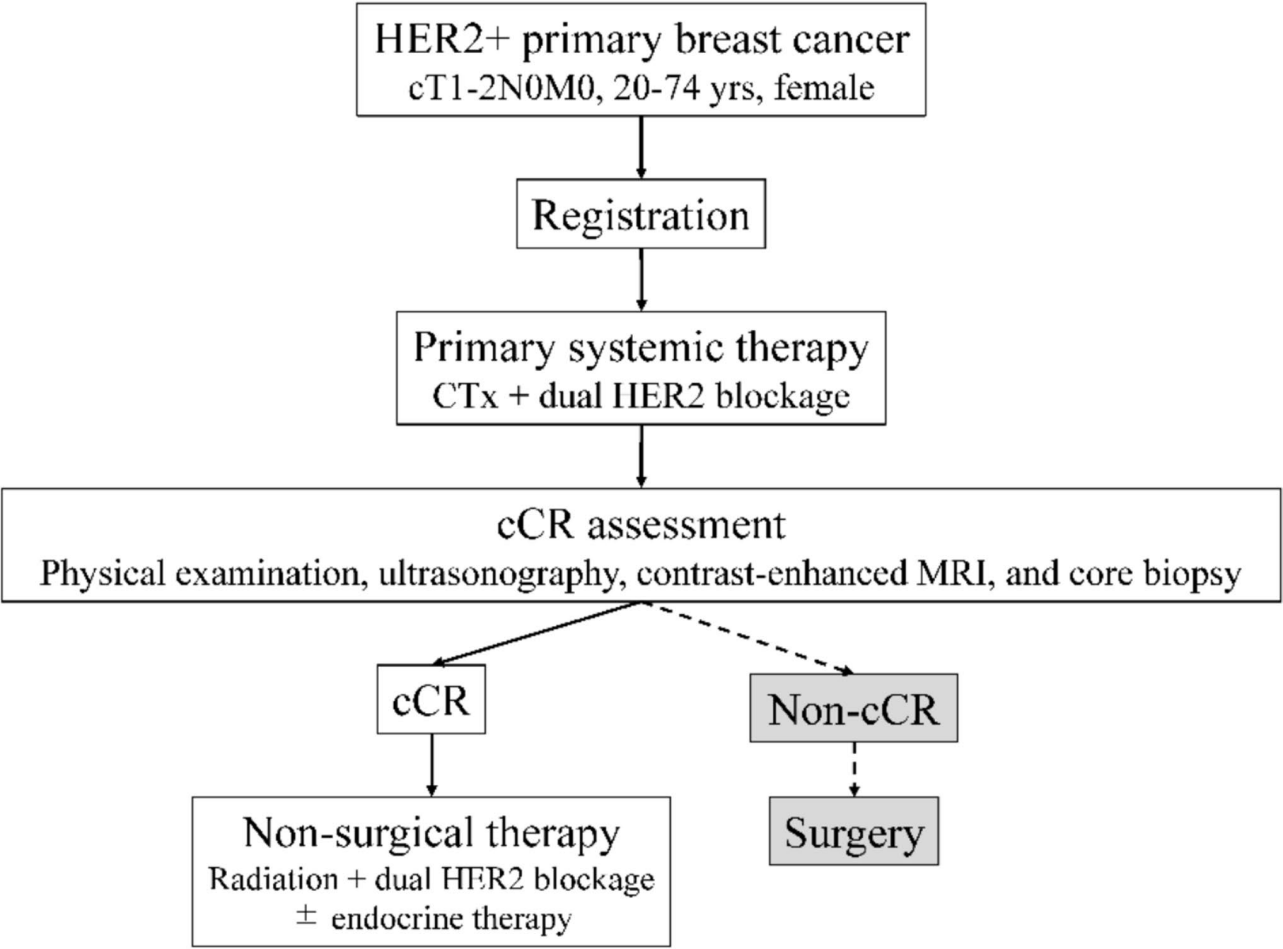
Study design of the JCOG1806 trial Core needle biopsy was performed to assess the cCR, optional for hormone receptor (HR)-negative breast cancer and mandatory for HR-positive breast cancer. HER2, human epidermal growth factor receptor 2; CTx, chemotherapy; cCR, clinical complete response; yrs, years; MRI, magnetic resonance imaging

This was an exploratory analysis of the ongoing JCOG1806 trial, a single-arm, confirmatory phase III study on the omission of surgery in patients with cT1–2N0M0, HER2-positive early breast cancer who exhibit a cCR after PST. In the JCOG1806 protocol, PST consisted of chemotherapy combined with dual HER2 blockade. Patients with a cCR are treated with radiotherapy and continued anti-HER2 therapy without surgery. The primary endpoint of the parent trial is 3-year distant metastasis-free survival, with final evaluation scheduled for March 2026. The trial is registered in the Japan Registry of Clinical Trials (jRCTs031190129), and the overall study design is shown in Fig. 1.

**Fig. 2:**
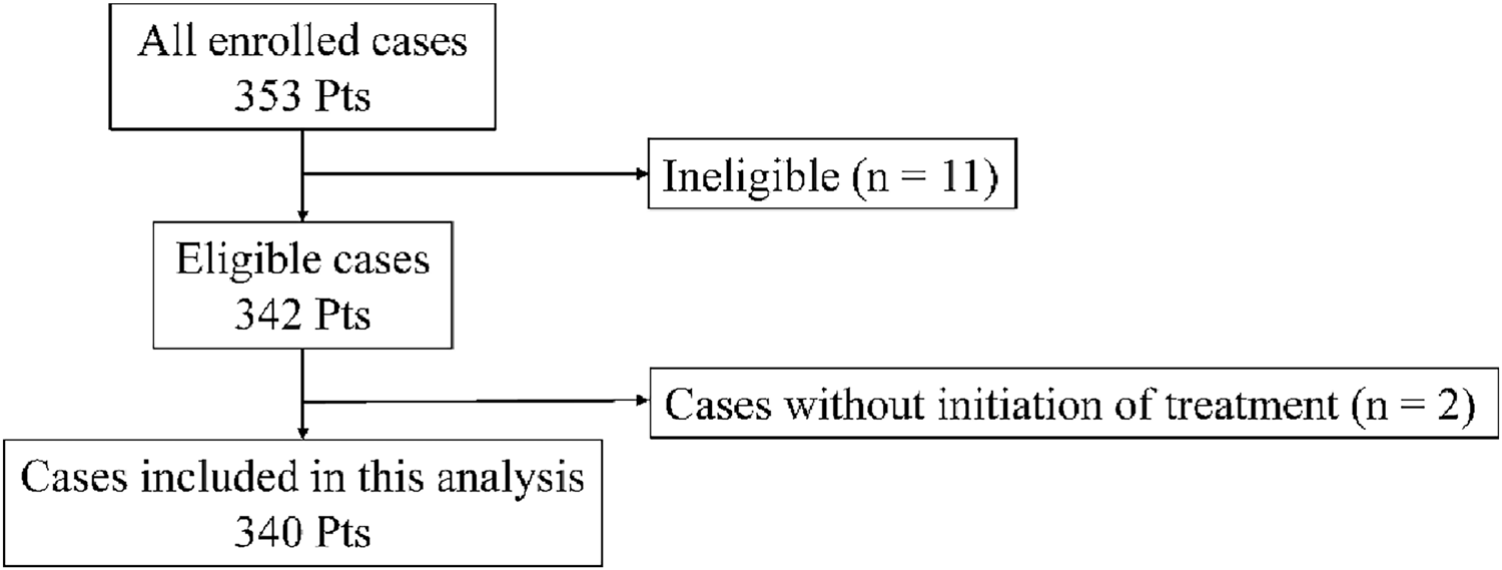
Patient flow of the exploratory analysis of patients from the JCOG1806 trial HER2, human epidermal growth factor receptor 2; CTx, chemotherapy; cCR, clinical complete response; Pts, patients

For the exploratory analysis, we included 340 patients from the JCOG1806 trial who were initiated on PST. Of the 353 patients enrolled between November 2019 and March 2023, 13 were excluded: nine due to ineligibility (including six without pre-registration assessments, two with BRCA mutations, and one with Li-Fraumeni syndrome), two because of incorrect staging after registration (lymph node metastasis or HER2-negativity), and two owing to non-initiation of PST (one patient declined treatment and one had hepatic dysfunction). This exploratory sub-analysis was conducted using data prospectively collected in the JCOG1806 trial and was performed within the scope of the original study protocol and institutional review board approval; therefore, no additional ethical approval or informed consent was required (Fig. 2).

### Eligibility criteria

To be eligible for JCOG1806, patients were required to be female, aged from 20 to 74 years, and have an Eastern Cooperative Oncology Group performance status of 0 or 1. They had to have histologically confirmed invasive, HER2-positive, primary breast cancer classified as cT1–2N0M0. HER2-positivity was defined as an immunohistochemistry (IHC) score of 3 + , an HER2/chromosome enumeration probe 17 amplification ratio ≥ 2.0 upon in situ hybridization (ISH), or a HER2 copy number of ≥ 6.0 signals per cell [17]. The ER and progesterone receptor (PR) statuses were also determined via IHC [18]; tumors with < 1% positive staining were considered ER- or PR-negative. Patients with tumors that were both ER- and PR-negative were classified as hormone receptor (HR)-negative. Imaging studies, including breast ultrasonography and contrast-enhanced MRI, were conducted to confirm that the primary tumor was 5.0 cm or smaller, with no evidence of multicentric lesions in the ipsilateral breast. The lymph node status was confirmed as negative based on imaging or histopathology. Additionally, distant metastasis was excluded using computed tomography (CT), bone scans, or fluorodeoxyglucose positron emission tomography–CT. Patients known to carry pathogenic BRCA1/2 mutations were excluded. Written informed consent was obtained from all the participants.

### Primary systemic therapy

One of the following three PST regimens was selected for each patient in this study, based on clinical factors. The first regimen consisted of sequential anthracycline-based chemotherapy (doxorubicin or epirubicin plus cyclophosphamide [AC/EC]) followed by a combination of a taxane (docetaxel or paclitaxel), trastuzumab, and pertuzumab (THP). The second regimen comprised docetaxel, carboplatin, trastuzumab, and pertuzumab (TCHP). The third regimen consisted of weekly paclitaxel with trastuzumab and pertuzumab (PacHP). AC/EC + THP and TCHP were administered to patients with either cT1 or cT2 tumors, whereas PacHP was administered only to patients with cT1 tumors. Detailed dosing schedules and cycle durations for each regimen are provided in Supplementary Table 1. Additional neoadjuvant treatment beyond the predefined PST regimens was not permitted under the study protocol. Before initiating PST, a marker was placed within the primary breast lesion to guide subsequent radiotherapy planning for patients for whom surgery was omitted. Chest CT was performed to confirm accurate marker placement.

### Criteria for definition of a cCR

A cCR was defined as the disappearance of the breast lesion. The definition of cCR differed according to HR status. For HR-negative, HER2-positive breast cancer, a cCR was defined as the absence of a palpable mass on physical examination and the absence of enhancing lesions on contrast-enhanced MRI. When scar tissue could not be distinguished from residual tumor tissue on breast ultrasonography, core needle biopsy was required to confirm the absence of malignancy. For HR-positive, HER2-positive cases, a cCR was similarly defined according to physical examination and MRI findings, but a core needle biopsy of the tumor bed was mandatory in all cases to confirm the absence of residual disease. The biopsy procedure—including needle gauge and number of cores—was left to the discretion of each participating institution. Histopathological evaluation of core needle biopsy specimens was performed at each participating JCOG-affiliated institution by board-certified diagnostic pathologists, and cases with residual invasive carcinoma or ductal carcinoma in situ (DCIS) were classified as non-cCR. Patients who exhibited disease progression during PST or discontinued treatment before cCR assessment were classified as having a non-cCR, and were withdrawn from the trial protocol accordingly.

### Surgical management for patients without a cCR

For patients who did not have a cCR after PST and in whom disease progression was observed during treatment, surgical intervention was performed. The type of breast surgery and axillary staging procedure were determined at the discretion of the attending physician. Pathological evaluation of the surgical specimens included assessment of the surgical margin, the ypT stage, the size of the largest invasive tumor, and the ypN stage.

### Statistical analysis

The primary objectives of this analysis were to evaluate the cCR rate after neoadjuvant therapy and to explore the potential predictive factors for a cCR. The cCR rate was defined as the proportion of eligible patients treated with PST who had a cCR. Univariate and multivariable logistic regression analyses were performed to assess the associations between a cCR and clinical variables—age, tumor size (cT stage), HR status, *HER2* expression level (IHC score), histological grade, and PST regimen. All variables were included in the multivariable model to adjust for potential confounders. Odds ratios (ORs) and 95% confidence intervals (CIs) were calculated for regression analyses. All statistical analyses were performed using SAS software (version 9.4; SAS Institute Inc., Cary, NC, USA).

## Results

### Patient characteristics

The baseline characteristics of the 340 included patients are summarized in Table 1. The median age was 56.0 (range, 27–74) years, with 49.6% aged ≤ 55 years. A majority of the patients were postmenopausal (64.4%). Regarding tumor size, 42.1% of patients had cT1 tumors and 57.9% had cT2 tumors. Most of the patients (95.6%) had invasive ductal carcinomas. The ER status was negative in 58.8% of patients, weakly positive (1%–9%) in 5.9%, and strongly positive (≥ 10%) in 35.3%. The PR status was negative in 70.9% of patients, weakly positive in 4.7%, and strongly positive in 24.4%. Among the 316 cases evaluated via IHC, *HER2* expression was 3 + in 88.3% of patients, 2 + in 11.4%, and 1 + in 0.3%. In total, 40.3% of the patients had histological grade III tumors. PST was discontinued for 11 patients (10 owing to adverse events and 1 at the patient’s request); those cases were categorized as having a non-cCR, per protocol.

**Table 1:** Patient characteristics

Factor		Value
Total		N = 340
Age, years	Median (Q1-Q3)	56 (48.5–63.0)
	< 55	169 (49.7%)
	> 56	171 (50.3%)
Menopausal status	Pre	120 (35.3%)
	Post	219 (64.4%)
	Unknown	1 (0.3%)
Clinical T stage	cT1	143 (42.1%)
	cT2	197 (57.9%)
Histological type	IDC NST	325 (95.6%)
	Special type	15 (4.4%)
ER	Negative	200 (58.8%)
	1%–9%	20 (5.9%)
	≥ 10%	120 (35.3%)
PR	Negative	241 (70.9%)
	1%–9%	16 (4.7%)
	≥ 10%	83 (24.4%)
HER2 (IHC)	0	0 (0%)
	1 + ^*^	1 (0.3%)
	2 +	36 (10.6%)
	3 +	279 (82.1%)
	Not tested (ISH only)	24 (7.1%)
Histological grade	I	27 (7.9%)
	II	176 (51.8%)
	III	137 (40.3%)
PST regimen	AC/EC + THP	296 (87.1%)
	TCHP	11 (3.2%)
	PacHP	33 (9.7%)

Q, Quartile; IDC NST, Invasive ductal carcinoma of no special type; ER, Estrogen receptor; PR, Progesterone receptor; HER2, Human epidermal growth factor receptor 2; IHC, Immunohistochemistry; ISH, In situ hybridization; PST, Primary systemic therapy; AC/EC, Doxorubicin/cyclophosphamide or epirubicin/cyclophosphamide; THP, Trastuzumab, pertuzumab, and taxane; TCHP, Docetaxel, carboplatin, trastuzumab, and pertuzumab; PacHP, Paclitaxel, trastuzumab, and pertuzumab

^*^ One patient with HER2 IHC 1 + was ISH-positive and classified as HER2-positive per protocol

### cCR rate and predictive factors

The overall cCR rate was 57.6% (196/340; 95% CI, 52.2–63.0%). The associations between patient characteristics and a cCR, based on logistic regression analyses, are presented in Table 2.

**Table 2:** Logistic regression analysis of the cCR rate

Factor		cCR rate	Univariate OR (95% CI)	*P* value	Multivariable OR (95% CI)	*P* value
Age, years	≤ 55	52.7% (89/169)	1	–	1	–
	≥ 56	62.6% (107/171)	1.50 (0.98–2.32)	0.065	1.40 (0.85–2.31)	0.18
Clinical T stage	cT1	61.5% (88/143)	1	–	1	–
	cT2	54.8% (108/197)	0.76 (0.49–1.18)	0.22	0.64 (0.38–1.10)	0.11
Histological type	IDC NST	57.5% (187/325)	1	–	1	–
	Special type	60.0% (9/15)	1.11 (0.39–3.18)	0.85	1.48 (0.47–4.61)	0.50
ER	Negative	67.5% (135/200)	1	–	1	–
	1%–9%	55.0% (11/20)	0.59 (0.23–1.49)	0.264	0.63 (0.22–1.81)	0.39
	≥ 10%	41.7% (50/120)	0.34 (0.22–0.55)	< 0.001	0.41 (0.20–0.81)	0.011
PR	Negative	64.3% (155/241)	1	–	1	–
	1%–9%	37.5% (6/16)	0.33 (0.12–0.95)	0.039	0.75 (0.22–2.56)	0.65
	≥ 10%	42.2% (35/83)	0.41 (0.24–0.67)	< 0.001	1.19 (0.55–2.58)	0.66
HER2(IHC)	1 + , 2 +	40.5% (15/37)	1	–	1	–
	3 +	60.2% (168/279)	2.22 (1.10–4.46)	0.025	2.19 (1.01–4.74)	0.046
Histological grade	I	29.6% (8/27)	1	–	1	–
	II	55.1% (97/176)	2.92 (1.21–7.01)	0.017	2.92 (1.07–7.93)	0.036
	III	66.4% (91/137)	4.70 (1.91–11.54)	0.001	4.90 (1.76–13.7)	0.002
PST regimen	AC/EC + THP	57.1% (169/296)	1	–	1	–
	TCHP	72.7% (8/11)	2.00 (0.52–7.70)	0.31	2.45 (0.58–10.4)	0.22
	PacHP	57.6% (19/33)	1.02 (0.49–2.11)	0.96	0.96 (0.40–2.31)	0.92

OR, Odds ratio; CI, Confidence interval; cCR, Clinical complete response; IDC NST, Invasive ductal carcinoma of no special type; ER, Estrogen receptor; PR, Progesterone receptor; HER2, Human epidermal growth factor receptor 2; IHC, Immunohistochemistry; PST, Primary systemic therapy; AC/EC, Doxorubicin/cyclophosphamide or epirubicin/cyclophosphamide; THP, Trastuzumab, pertuzumab, and taxane; TCHP, Docetaxel, carboplatin, trastuzumab, and pertuzumab; PacHP, Paclitaxel, trastuzumab, and pertuzumab

The cCR rate was 67.5% among patients with ER-negative tumors and 41.7% among those with strongly ER-positive tumors. Regarding *HER2* expression, 60.2% of patients with IHC 3 + tumors had a cCR compared to the rate of 40.5% among those with IHC 1 + or 2 + tumors. The cCR rates were 66.4%, 55.1%, and 29.6% among patients with histological grade III, II, and I tumors, respectively.

Upon multivariable analysis, strongly ER-positive tumors were less likely to have a cCR compared than ER-negative tumors (OR, 0.41; 95% CI, 0.20–0.81; *p* = 0.011). Tumors with an IHC score of 3 + were associated with higher odds of a cCR than those with an IHC score of 1 + or 2 + (OR, 2.19; 95% CI, 1.01–4.74; *p* = 0.046). Compared with histological grade I tumors, the odds of a cCR were higher for grade II (OR, 2.92; 95% CI, 1.07–7.93; *p* = 0.036) and III (OR, 4.90; 95% CI, 1.76–13.7; *p* = 0.002) tumors.

### Surgical and pathological findings in Non-cCR cases

Among the 136 patients classified as non-cCR, 126 underwent surgery (Table 3). Breast-conserving surgery was performed in 80 patients (63.5%), whereas mastectomies were performed in 46 (36.5%). Axillary staging was conducted in 124 cases, including sentinel lymph node biopsy in 116 and axillary lymph node dissection in 8 patients. The ypT stage was ypT0 in 28 cases (22.2%), ypTis in 25 cases (19.8%), and ypT1 or higher in 73 cases (57.9%). Thus, 22.2% of patients with a non-cCR had no residual invasive or in situ carcinoma in the breast upon pathological examination. The median diameter of residual invasive tumors was 2.8 mm (quartiles 1–3, 0.0–10.0 mm). Lymph node metastases were observed in 10 of the 126 patients (7.9%), including four with micrometastases (ypNmi).

**Table 3:** Surgical and pathological findings in non-cCR cases

Factor		Value
Total		*N* = 126
Breast surgery	Breast-conserving surgery	80 (63.5%)
	Mastectomy	46 (36.5%)
Axillary staging Surgical margin	None	2 (1.6%)
	Sentinel lymph node biopsy	116 (92.1%)
	Axillary lymph node dissection	8 (6.3%)
	Negative	116 (92.1%)
	Positive	10 (7.9%)
ypT stage	ypT0	28 (22.2%)
	ypTis	25 (19.8%)
	ypT1	63 (50.0%)
	ypT2	10 (7.9%)
Size of invasion (mm)	Median	2.8
	Q1-Q3	0.0–10.0
	Min–Max	0.0–31.0
ypN stage	ypN0	116 (92.1%)
	ypNmi	4 (3.2%)
	ypN1	6 (4.8%)

cCR, Clinical complete response; Q, Quartile

## Discussion

In this exploratory analysis of the JCOG1806 trial, we evaluated the clinical outcomes and predictive factors for achieving a cCR in patients with HER2-positive early breast cancer. The cCR rate following PST was 57.6%. An ER-negative status, IHC score of 3 + , and histological grade III were identified as independent predictors of a cCR. Furthermore, 22.2% of patients with a non-cCR who underwent surgery had a ypT0 status upon pathological examination, indicating the potential for misclassification under current criteria. These findings provide a basis for refinement of patient selection in strategies aimed at omitting surgery in this disease setting.

In this exploratory analysis of the JCOG1806 trial, 57.6% of patients with HER2-positive early breast cancer achieved a cCR and were therefore considered eligible for omission of surgery. The cCR rates across ER subgroups—67.5% in ER-negative, 55.0% in weakly ER-positive, and 41.7% in strongly ER-positive tumors—were comparable to previously reported pCR rates in patients treated with dual HER2 blockade-based PST [5, 7, 19], supporting the validity of the cCR assessment criteria used in this trial. In addition, this study identified an IHC score of 3 + and a higher histological grade as independent predictors of a cCR. The association between IHC 3 + status and treatment response is consistent with prior reports linking strong HER2 overexpression to higher pCR rates [20, 21]. Similarly, the CTNeoBC pooled analysis demonstrated a strong association between higher histological grade and pCR [1]. Taken together, these findings suggest that clinicopathological features such as ER negativity, an IHC score of 3 + , and grade III histology may help identify patients most likely to benefit from the omission of surgery after PST.

In the context of surgery-omission strategies after PST, accurate prediction of pCR is essential for identifying appropriate candidates. Previous studies have shown that an MRI-defined cCR is strongly associated with pCR and demonstrates particularly high concordance in HER2-positive breast cancer, with prognostic value comparable to that of pCR [22–25]. Image-guided core needle biopsy after neoadjuvant chemotherapy has also been reported to improve diagnostic accuracy for confirming residual disease [26–28]. In the JCOG1806 protocol, cCR was defined using a combined assessment based on contrast-enhanced MRI and core needle biopsy, aiming to ensure accurate prediction of pCR while maintaining applicability to clinical practice. Disappearance of the lesion on MRI was used as the primary criterion, with additional biopsy performed according to HR status. Needle biopsy was required for HR-positive disease, in which concordance between cCR and pCR is known to be lower, whereas biopsy was omitted for HR-negative disease, where higher concordance has been consistently reported [24, 25].

In the JCOG1806 trial, 22.2% of patients classified as non-cCR were found to have ypT0 disease on surgical pathology, indicating that reducing the false-positive rate in cCR assessment is an important challenge for surgery-omission strategies. In this trial, cCR was primarily determined by the disappearance of the lesion on contrast-enhanced MRI. However, previous studies have shown that MRI after NAC yields a certain proportion of false-positive findings, highlighting a limitation of imaging-based assessment in identifying candidates for surgery omission [22, 29]. In a prior trial evaluating surgery omission, favorable outcomes have been reported even in patients with minimal residual findings on imaging when a complete response was confirmed by image-guided biopsy [11, 12]. These findings suggest that combining imaging and biopsy assessments may better identify candidates for surgery omission and help avoid unnecessary surgery in patients who have already achieved tumor eradication.

This study had several limitations. First, the study population was limited to patients with cT1–2N0M0 HER2-positive breast cancer, which may restrict the generalizability of the findings to patients with more advanced disease (e.g., cT3 or node-positive cases). Second, although the flexible biopsy approach was intended to reflect real-world practice, the absence of standardized procedural guidelines might have affected diagnostic consistency. Third, MRI interpretation was performed locally without central review, and inter-observer agreement was not evaluated, which might have introduced variability in cCR determination. In addition, long-term outcomes of patients who achieved cCR in the JCOG1806 trial have not yet been clarified, and results from future analyses are awaited.

## Conclusions

In this exploratory analysis of the JCOG1806 trial, 57.6% of patients with HER2-positive early breast cancer had a cCR after PST, with ER-negativity, an HER2 IHC score of 3 + , and higher histological grade being identified as predictive factors. These findings offer valuable insights for the optimization of treatment strategies regarding the omission of surgery in this patient population.

## Ethics approval and consent to participate

All methods were performed in accordance with relevant guidelines and regulations. This study was approved by the Certified Review Board of the National Cancer Center Hospital East, and the approval reference number is CRB3180009. Written informed consent was obtained from all participants before enrollment.

## Consent to publish

Not applicable.

## Supplementary Information

Below is the link to the electronic supplementary material.

Supplementary file1 (DOCX 15 kb)

## Data Availability

Individual participant data that underlie the results reported in this article will not be shared because patient follow-up will continue until May 2028. After the publication of the data as of May 2029, de-identified participant data that underlie the results will be shared to investigators upon approval of their proposed use of the data by the investigators of the Breast Cancer Study Group of the JCOG. Proposals should be sent to shigematu1330@yahoo.co.jp. The data will be made available only to achieve the aims of the approved proposal.
