# Supplementary material for: Clinical complete response and predictive factors in HER2-positive early breast cancer treated with neoadjuvant chemotherapy aimed at omission of surgery: an exploratory analysis of the JCOG1806 trial
Source: Int J Clin Oncol. 2026 Jan 22;31(3):528–36. doi: 10.1007/s10147-026-02967-7 (PMC12932372; doi:10.1007/s10147-026-02967-7)
Supplement: Supplementary file 1 — Supplementary file1 (DOCX 15 kb) [file 10147_2026_2967_MOESM1_ESM.docx]

Supplementary Table 1. Neoadjuvant Chemotherapy Regimens in the JCOG1806 Trial

Trastuzumab: 8 mg/kg loading dose, then 6 mg/kg every 3 weeks. Pertuzumab: 840 mg loading dose, then 420 mg every 3 weeks. Patients who had previously received systemic therapy for another malignancy and had a cumulative anthracycline dose exceeding 260 mg/m² for doxorubicin or 540 mg/m² for epirubicin, or an unknown total dose, were assigned to the TCHP regimen.

| **Regimen** | **Component Drugs** | **Dose and Schedule** | **Number of Cycles** | **Applicable Tumor Stage** |
| --- | --- | --- | --- | --- |
| **AC/EC → THP** | Doxorubicin or Epirubicin + Cyclophosphamide (AC or EC) | Doxorubicin 60 mg/m² or Epirubicin 90 mg/m² + Cyclophosphamide 600 mg/m², every 3 weeks | 3–4 cycles | cT1 or cT2 |
|  | Docetaxel or Paclitaxel + Trastuzumab + Pertuzumab (THP) | Docetaxel 75 mg/m² every 3 weeks or Paclitaxel 80 mg/m² weekly + Trastuzumab + Pertuzumab | 3–4 cycles |  |
| **TCHP** | Docetaxel + Carboplatin + Trastuzumab + Pertuzumab | Docetaxel 75 mg/m² + Carboplatin (AUC 6) + Trastuzumab + Pertuzumab, every 3 weeks | 5–6 cycles | cT1 or cT2 |
| **PacHP** | Paclitaxel + Trastuzumab + Pertuzumab | Paclitaxel 80 mg/m² weekly + Trastuzumab + Pertuzumab, every 3 weeks | 3–4 cycles | cT1 only |
